# Pattern recognition of spectral entropy features for detection of alcoholic and control visual ERP’s in multichannel EEGs

**DOI:** 10.1007/s40708-017-0061-y

**Published:** 2017-01-21

**Authors:** T. K. Padma Shri, N. Sriraam

**Affiliations:** 1 Department of Electronics and Communication, Manipal Institute of Technology, Manipal University, Manipal, Karnataka 576104 India; 2 Department of Medical Electronics, M.S. Ramaiah Institute of Technology (An Autonomous Institute, Affiliated to Visvesvaraya Technological University), Bangalore, Karnataka 560054 India

**Keywords:** Visual event-related potentials (visual ERP), Electroencephalogram (EEG), Spectral entropy (SE), Gamma sub-band, Principal component analysis (PCA), Principal components (pcs), k-Nearest neighbor (k-NN) classifier

## Abstract

This paper presents a novel ranking method to select spectral entropy (SE) features that discriminate alcoholic and control visual event-related potentials (ERP’S) in gamma sub-band (30–55 Hz) derived from a 64-channel electroencephalogram (EEG) recording. The ranking is based on a *t* test statistic that rejects the null hypothesis that the group means of SE values in alcoholics and controls are identical. The SE features with high ranks are indicative of maximal separation between their group means. Various sizes of top ranked feature subsets are evaluated by applying principal component analysis (PCA) and k-nearest neighbor (k-NN) classification. Even though ranking does not influence the performance of classifier significantly with the selection of all 61 active channels, the classification efficiency is directly proportional to the number of principal components (pc). The effect of ranking and PCA on classification is predominantly observed with reduced feature subsets of (*N* = 25, 15) top ranked features. Results indicate that for *N* = 25, proposed ranking method improves the k-NN classification accuracy from 91 to 93.87% as the number of pcs increases from 5 to 25. With same number of pcs, the k-NN classifier responds with accuracies of 84.42–91.54% with non-ranked features. Similarly for *N* = 15 and number of pcs varying from 5 to 15, ranking enhances k-NN detection accuracies from 88.9 to 93.08% as compared to 86.75–91.96% without ranking. This shows that the detection accuracy is increased by 6.5 and 2.8%, respectively, for *N* = 25, whereas it enhances by 2.2 and 1%, respectively, for *N* = 15 in comparison with non-ranked features. In the proposed *t* test ranking method for feature selection, the pcs of only top ranked feature candidates take part in classification process and hence provide better generalization.

## Introduction

Alcoholism is a chronic disease that is addictive and progressive in nature. There are genetic, environmental, and psychosocial factors which determine the extent to which alcoholism turns into alcohol abuse. A lot of studies have shown the ill effects of alcoholism on various organs of the body, especially on the brain [1–6]. Prefrontal dysfunction in alcoholics is well understood by a lot of studies [7]. Alcohol consumption releases dopamine into nucleus accumbens and prefrontal cortex which is hypothesized to reinforce drinking habit [8]. Studies have also revealed that the alcohol consumption affects the non-alcoholic offspring of alcoholic parents [9, 10]. One of the simplest and cost-effective tools to study the real-time effects of alcoholism is the EEG recorded on the scalp of human brain. While recording the EEG with an internal or external stimulus, ERPs exhibit cerebral activity that characterizes the spatiotemporal changes in the human brain over a period of time due to alcoholism [4, 5, 11]. These changes persist even after long-term abstinence from alcohol [12]. The dynamic processes of the brain such as memory, attention, and cognitive processing [13, 14] are correlated with the synchronizations of phase-locked peaks generated by ERPs. These dynamic processes exhibit themselves at different frequencies known as the delta, theta, alpha, beta, and gamma waves. Changes in the characteristics of these waves due to alcoholism are also reported by a lot of studies [15–17].

Studies on visual ERPs of alcoholics [5, 13, 18–21] have reported that there is a reduction in evoked gamma oscillations during the processing of a visual object recognition task. As these oscillations are correlated with cognition processes such as selective attention and working memory, there is a need to identify specific regions of brain that are largely influenced by alcohol consumption during these event-related oscillations. Therefore, identification of channels with high discrimination between alcoholic and control groups using features extracted from visual ERP’s of a multichannel EEG recording in gamma band needs to be investigated. Feature subset selection from 64-channel EEG recordings of alcoholics and controls has been reported [22–25] in literature. All the above studies use the same alcohol EEG dataset used in the current study with 30 alcoholic and 30 control subjects visual ERP’s. In one of these studies [22] seven out of 61-channel EEG are selected based on genetic algorithm (GA) optimization to effectively discriminate alcoholics and controls, providing an average classification accuracy of 94.3 and 81.8% with multilayered perceptron-back propagation (MLP-BP) network and fuzzy art map (FA) classifier, respectively. Studies in [23] have shown that the use of PCA for reducing the number of channels resulted in classification accuracies of 95.83, 94.06, 86.01, and 75.13% for 61, 16, 8, and 4 channels, respectively. The correlation between the selected optimal subset of channels for alcoholics was explored in a study [24] to select the channel subset based on the mean gamma band power. The vectors consisting of mean gamma band power from highly correlated channels were used to train a least-square SVM classifier to discriminate alcoholics from their control counterparts. An average classification accuracy of 80% was reported among different pairs of retained active channels. Another study [25] reported using nonlinear parameters such as ApEn, sample entropy, Lyapunov exponent, and higher-order spectra for feature extraction and detection of alcoholics with maximum accuracy of 91.7% with support vector machine (SVM) classifier. The channel selection in this study is based on statistical *t* test and only seven statistically significant channels were used for classification. In all these studies, even though the selection of a subset of channels is based on certain criterion, the merit of each of the individual channels within the subset is not weighed in terms of its ability to separate alcoholics from controls. Also, the selected channels are not correlated with the position on the scalp. Hence the main objective of the proposed research is not only to explore the possibility of identifying and retaining those channels which show more dissimilar activity in the visual ERPs of alcoholics and controls but also to rank them in terms of their ability to discriminate the groups. The novelty of this study lies in ranking and reducing the dimensionality of multichannel EEG data using *t* test and PCA for the localization of visual ERPs of alcoholics and controls groups. In the proposed study, the spectral entropies of 61 active (three reference electrodes) channels in the gamma sub-band constitute the feature vector for each subject and are used as features to classify alcoholics and controls. The extracted SE features are ranked using statistical *t* test analysis and the dimensionality of the ranked feature vector is reduced by applying PCA. The validity of the proposed ranking method in identifying channels of high significance (as far as the effect of alcohol on visual ERPs in these regions is concerned) is evaluated by applying the ranked and reduced feature set as inputs to k-NN classifier. For a predetermined set of ranked channels (*N* = 61, 25, 15), PCA is performed and a subset of pcs are chosen from these ranked channels to evaluate the performance of a k-NN classifier for pattern recognition. This method is repeated for a set of non-ranked features of the same size. The order of non-ranked channels is according to those specified in the EEG dataset. The classifier results are cross-validated using hold out cross-validation.

The rest of the paper is organized as follows: Sect. 2 presents the methodology and implementation. Section 3 presents results and discussions, followed by conclusions and future work in Sect. 4.

## The proposed method

In our earlier work [26–29], despite obtaining good classification accuracy, the channels which contribute to the discrimination of alcoholics were not identified. In our recently published work [21], a statistical measure called SEPCOR is used to rank the features and classification results are very impressive. In order to further evaluate statistically significant features, ranking and reduction in SE features are performed on the visual ERPs of alcoholics/controls using *t* test and PCA in the proposed study for studying the impact of alcohol on specific regions of the brain.

**Fig. 1:**
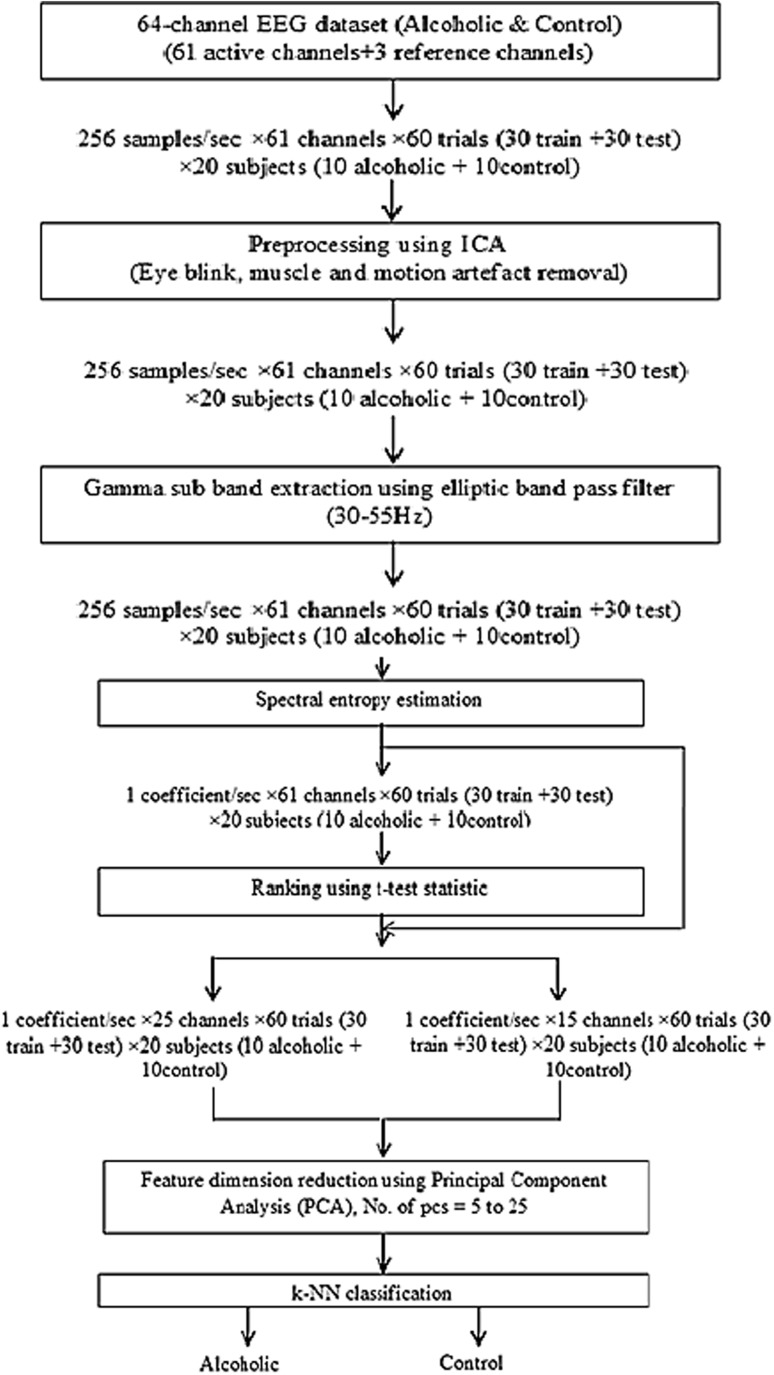
Schematic of the proposed method

Figure 1 shows the proposed schematic flow.

### EEG data

The EEG dataset is from an open EEG Database of State University of New York Health Centre. These data arise from a large number of studies to examine EEG correlates of genetic predisposition to alcoholism. It contains measurements from 64 (61 active channels + 3 reference channels) electrodes placed on subject’s scalps which were sampled at 256 Hz [30]. The database consists of 64 channel EEG recordings of ten alcoholic and ten control subjects while performing a visual object recognition task. The picture objects were chosen from the 1980 Snodgrass and Vander wart picture set [31]. A single object S1 or two objects S1 and S2 were used as visual stimulus to each of the subjects both in S1–S2 matched condition and in S1–S2 unmatched condition. Ten trials were conducted in each condition for acquiring train set and the test set used the same ten alcoholic and ten control subjects, but with ten out-of-sample runs per subject per paradigm. This accounts for a total of 600 visual ERP patterns for the training data and 600 visual ERP patterns for the testing data, each of them lasting for a second.

### Data preprocessing

1. Eye blink artifact removal and gamma sub-band extraction

Eye blink artifact produces a 100–200-µV potential lasting for 250 ms [32]. Some of the other sources of artifact consist of surface muscle activity (>30 Hz), body movement, etc. Independent component analysis (ICA) is performed on the entire EEG dataset to separate these artifacts to obtain artifact-free EEG epochs for further processing [21].

Studies have reported that early phase-locked gamma is evoked in selective attention and larger in response to attended stimuli than unattended stimuli, particularly in frontal lobe [19, 20]. It is also observed that the visual feature binding process is synchronized with the gamma band [18, 32]. Due to this reason, an elliptic band pass filter of sixth-order is used to extract gamma sub-band range of 30–55 Hz. The phase distortions caused due to filtering are compensated by applying the filter in forward/reverse direction.

### Spectral entropy feature extraction

Entropy estimation provides a measure of disorderliness, and hence some important information regarding the complexity of the processes involved in a system is obtained. The more the disorderliness (complexity) is, the higher is the entropy value. Neurophysiological evidence shows that as the cortex becomes unconscious, there is a true decrease in entropy occurring at the neuronal level [33]. Recently, entropy estimation of EEG signals has been used to explain how the EEG signals change with time either in frequency or in phase domain [34–37]. The change in information entropy within the EEG may reflect a real-time information transfer within the cortex.

Spectral entropy (SE) computation uses Shannon’s entropy formula to represent the power spectral densities as probabilities. Accordingly, the normalized SE corresponding to the frequency range [*f*_1_, *f*_2_] is calculated from 1-s epochs of 61-channel visual ERP’s of alcoholic and control group as follows:

1 $${\text{SE}}\left[ {f_{1} ,f_{2} } \right] = - \frac{1}{{\log \left[ {N\left[ {f_{1} ,f_{2} } \right]} \right]}}\mathop \sum \limits_{{f_{{i = f_{1} }} }}^{{f_{2} }} P_{n} \left( {f_{i} } \right)\log \left( {P_{n} \left( {f_{i} } \right)} \right)$$

where *P*_*n*_(*f*_*i*_) represents the probability of the *i*th frequency component. The step-by-step computation is explained in our earlier published work [21].

**Fig. 2:**
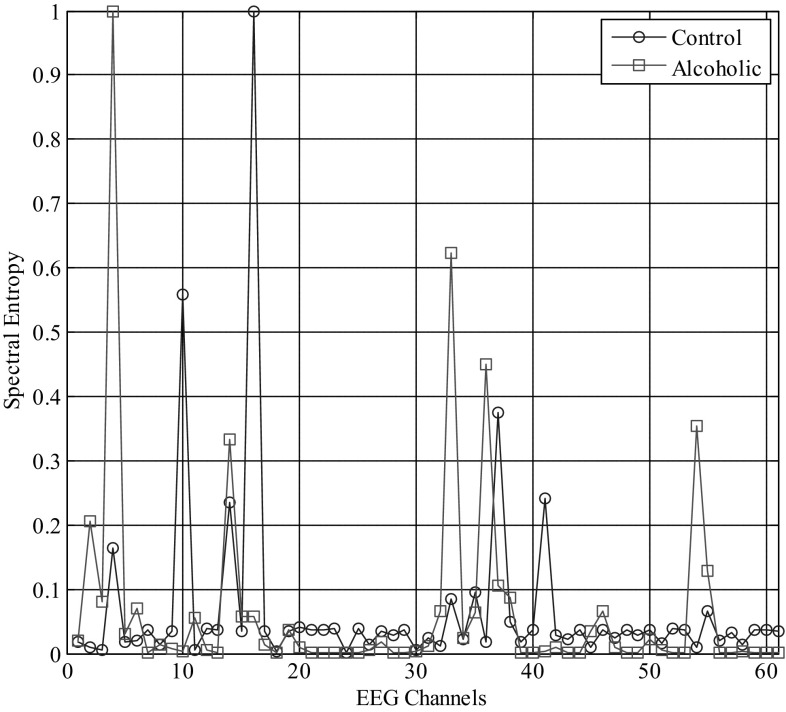
Spectral entropy plot of a single alcoholic/control subject

**Fig. 3:**
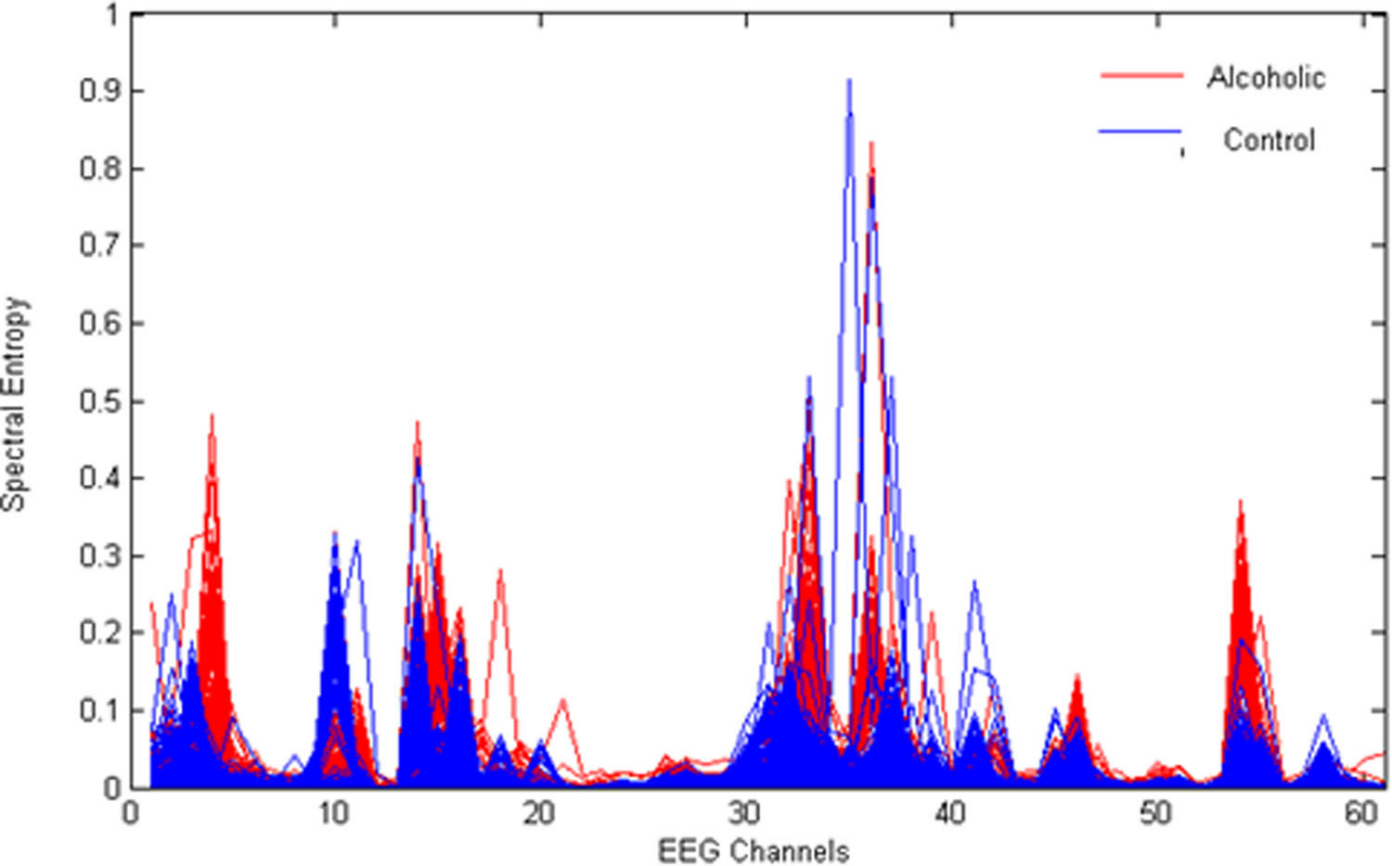
Plot of spectral entropy features for the entire dataset [21]

Each 1-s, 61-channel EEG data epoch (61 channels × 256 samples/s) is represented by a 61-component SE vector (61 × 1), called SE feature vectors. The entire feature set dimension is equal to (61 × 1200) corresponding to a total 1200 (test + train) visual ERP’s comprising of both alcoholics and controls. Figure 2 shows a sample plot of SE in all 61 channels for a single alcoholic and a single control subject, and Fig. 3 represents the SE plot for all 61 channels of the entire dataset consisting of 600 visual ERP’S of alcoholics and 600 ERP’s of controls. It is seen that in some channel locations (for example, 4 and 10), the gamma sub-band SE feature is more discriminative between groups, whereas in channel locations between 4 and 10, there is no difference in the computed SE feature for both groups. This suggests that only in certain locations of the scalp, the SE feature being a measure of complexity differs between Alco/control groups, while in other channel locations the complexity measure is not distinctive. This can be observed in other channel locations as well, leading to a very important conclusion that those channels with highly discriminative SE measures between groups identify themselves as better candidates for feature classification task. A detailed discussion of the same has been published by the same authors in [21].

### Ranking by *t* test and PCA

Spectral entropy estimation is applied to extract features from the visual ERP responses of alcoholics and controls. Next, the SE values of each channel are ranked based on their ability to discriminate alcoholics from their control counterparts by using hypothesis (*t* test) testing method (Eq. 2). The suitability of data for *t* test is determined by fitting the entire 61-channel SE data to normal distribution, and goodness of fit is evaluated using Kolmogorov–Smirnov test. Since the proposed classification problem has a single outcome variable of either detecting an alcoholic or non-alcoholic and data are parametric in which the population parameter is specified [38], the selection of independent *t* test is justified for feature ranking. The Welch two-sample *t* test is considered for analysis which is defined as (2):

2 $$t = \frac{{\left( {\bar{x}_{1} - \bar{x}_{2} } \right)}}{{\sqrt {\left( {{\raise0.7ex\hbox{${S_{1}^{2} }$} \!\mathord{\left/ {\vphantom {{S_{1}^{2} } {n_{1} }}}\right.\kern-0pt} \!\lower0.7ex\hbox{${n_{1} }$}} + {\raise0.7ex\hbox{${S_{2}^{2} }$} \!\mathord{\left/ {\vphantom {{S_{2}^{2} } {n_{2} }}}\right.\kern-0pt} \!\lower0.7ex\hbox{${n_{2} }$}}} \right)} }}$$

where $\bar{x}_{1} ,\,\bar{x}_{2}$ are the sample means, $s_{1}^{2} ,\,s_{2}^{2}$ are the sample variances and *n*_1_, *n*_2_ are the sample sizes of groups 1 (alcoholic) and 2 (control), respectively.

**Fig. 4:**
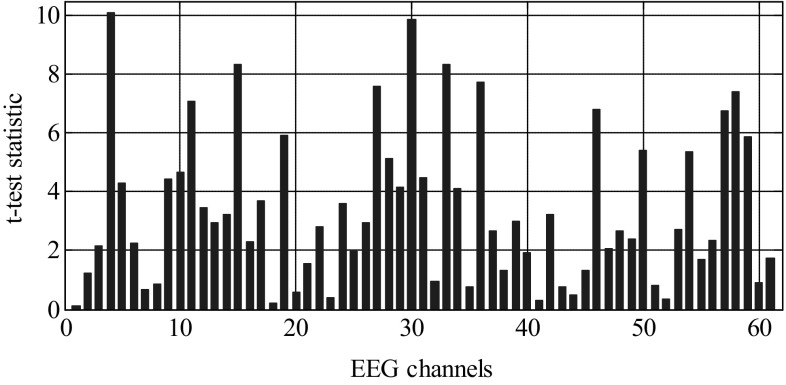
Plot of *t* test statistic for alcoholic and control SE feature vectors

**Fig. 5:**
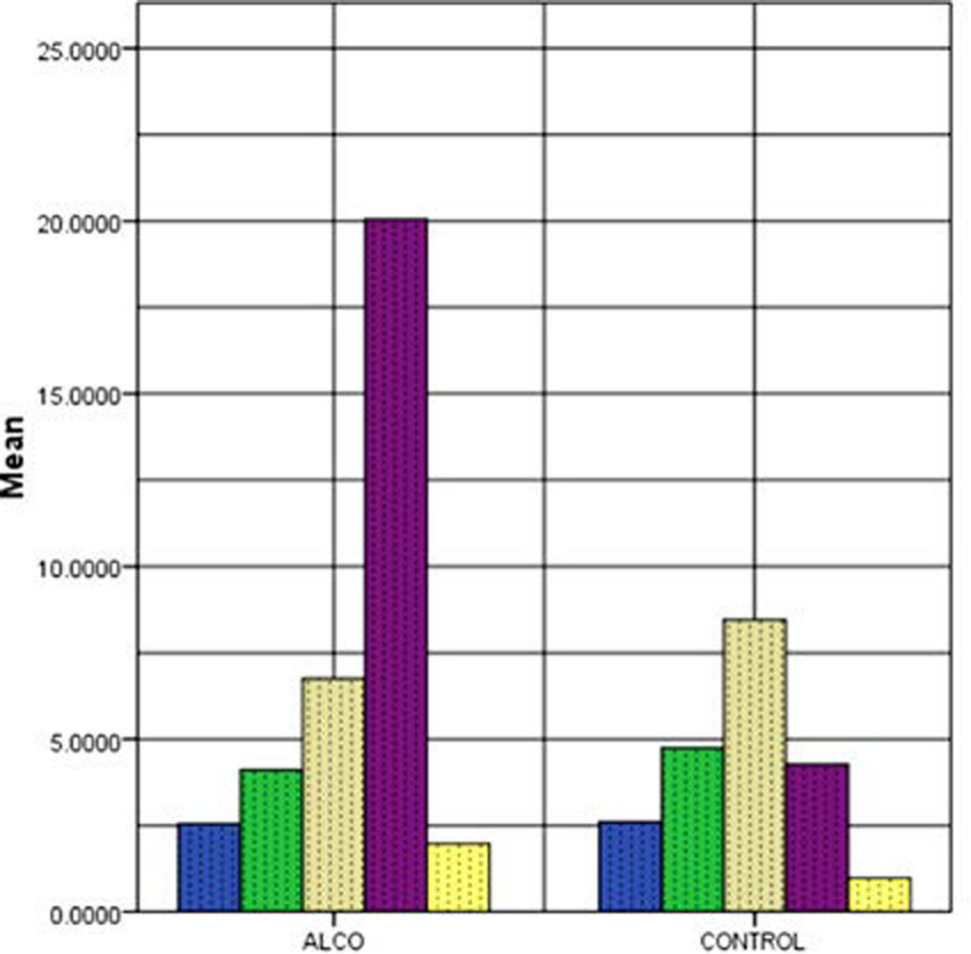
Bar plot of SE mean values in arbitrarily selected channels for alcoholic/control subjects

The *t* test statistic plot in Fig. 3 gives information regarding the difference in class means of SE feature for each channel. The ranking of channels is not only based on the statistical significance (*p* value) but also on the difference in class means, i.e., larger the difference in class means, higher the ranking for that channel. For example, from Fig. 4, it is seen that channels such as 4, 12, 15, 19, and 30, possess *t* statistic values that maximize the class separation than other channels with lower statistic. As an example, Fig. 5 shows the bar plot of SE mean for the first five channels of both alcoholic and control groups. The fourth channel has the largest difference in SE mean and hence is chosen as a candidate with higher ranking. Similarly, first channel scores lowest ranking among these channels as the difference in class means is zero. This procedure is actually performed on all 61 channels, and each channel is ranked based on the differences in class means. This ensures that it is not only sufficient for a channel to have *p* value <0.05, but the difference in class means must also be higher to achieve a higher ranking. The channels are ranked based on the statistically significant values (*p* < 0.05) obtained by performing *t* test on 61 channels of both groups with 95% confidence interval.

The ranking method assigns highest ranks to those channels in which the *p* values are lesser than 0.05. Higher ranks indicate that the visual ERP signals in these locations are largely influenced by the consumption of alcohol as compared to the control groups. The regions where the SE features score less ranking are less discriminative of both groups.

#### Principal component analysis (PCA)

In order to reduce the dimensionality of the features further and to study the effect of pcs, PCA is applied on both ranked and non-ranked channels.

*Implementation steps*

The aim here is to transform a *N* × n matrix *X* into a *N* × d matrix *Y*, where *X* represents the original SE feature space of dimension *N* × n, *N* represents the number of samples (1200), *n* represents the number of channels (61), *d* represents the feature subspace (arbitrarily selected as, 25 and 15), and *Y* represents the PCA-transformed feature matrix. The steps involved are:

- Subtract the mean of SE values from each channel variables of matrix, *N* × n (shifting the data to origin).
- Compute the covariance matrix *V*, of *n* × *n*
  Dimension as below:
  3 $$V = \frac{1}{N - 1}X^{T} X;$$
  i.e.,
  4 $$V_{i,j} = \frac{1}{N - 1}\mathop \sum \limits_{k = 1}^{N} X_{k,i} X_{k,j}$$
  The diagonal elements of *V* represent the variance of variable *i*, whereas the off-diagonal elements represent the covariance between *i* and *j*.
- Compute the eigenvectors of covariance matrix, *V*.
- Identify *d* eigenvectors that correspond to the largest *d* eigenvalues to be the new basis for the transformed data space.

### k-NN classification

In order to validate the results obtained by the proposed ranking method, the ranked and non-ranked SE feature vectors are applied as inputs to k-NN classifier. The k-nearest neighbor algorithm (k-NN) is a supervised nonparametric classifier [39, 40] used to classify patterns based on closest training pattern vectors in the feature space. The purpose of selecting k-NN classifier over other robust classification methods such as SVM and MLP is to make a preliminary evaluation of the ranked features. The advantage of k-NN classification is that it is a nonparametric method where no assumption on the distribution of data is made and that it is simple to implement. However, it is sensitive to local structure of the dataset. To address this problem, a 50% holdout cross-validation is performed. Initially, all 61 ranked features are applied to k-NN classifier, and the performance is evaluated in terms of classification accuracy and computational time. The classifier performance is evaluated by applying predetermined sets of ranked and non-ranked features (arbitrarily selected as 61, 25, and 15, respectively) with different number of pcs (randomly chosen values of 61, 25 and 15). For each of these cases, the discriminatory behavior of classifiers is studied and compared. The k-NN classifier algorithm is implemented on MATLAB platform.

## Results

Gamma deficits manifest themselves as cognitive deficits in selective attention and working memory of alcoholics [19, 20].Deficits in gamma band power to target stimuli in alcoholics, particularly in the frontal lobe, is well documented by a lot of studies [5, 21].

**Fig. 6:**
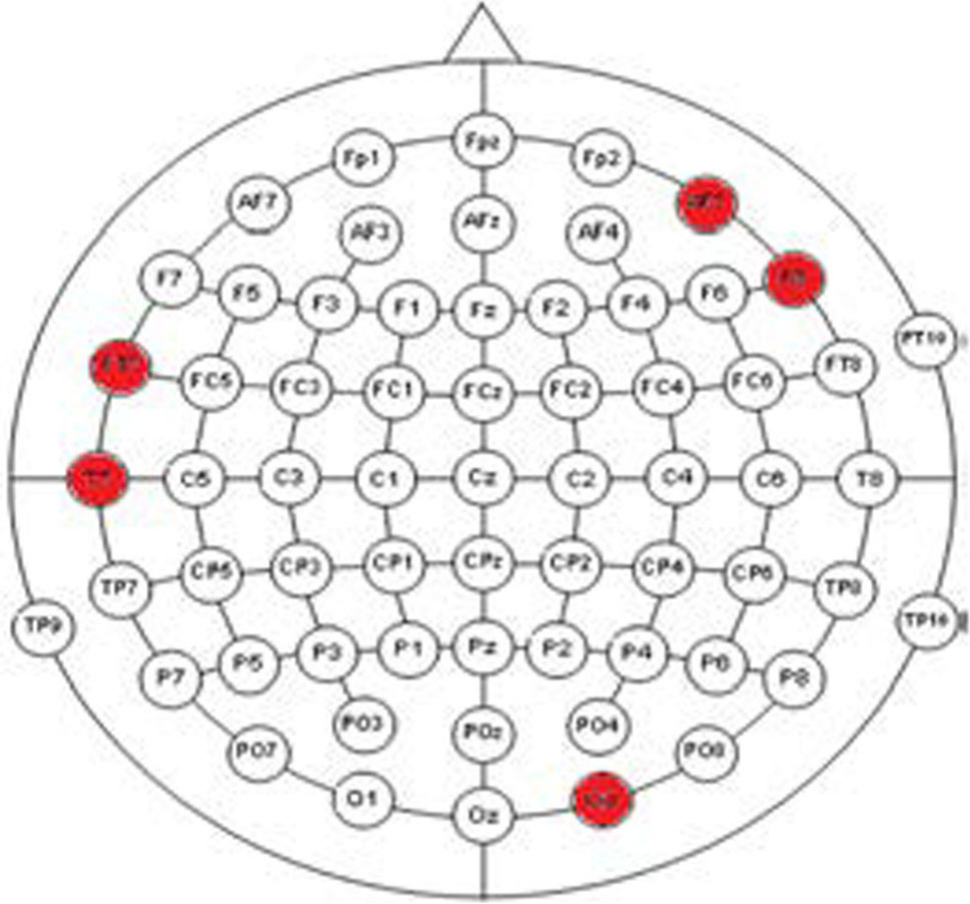
Active electrode positions (in *red*) with first five ranks. (Color figure online)

The SE features plotted for the entire dataset (Fig. 3) consisting of alcoholic and control subjects indicate the apparent differences in spectral entropies for alcoholics and controls in some channels. It is seen that the alcoholic SE values exceed in many of the locations on the scalp as compared to controls, indicating more complexity (disorderliness) in these regions. Thus the SE values reflect a measure of complexity in specific regions of the brain directly correlating with the difference in cognitive and information processing of both groups. To identify those regions in which alcoholics have specific cognitive and working memory deficits, ranking of features is performed based on a statistical (*t* test) hypothesis testing. The criterion for ranking 61-channel SE coefficients is based on a two-sample *t* test performed on both the groups and the resulting statistics are plotted in Fig. 4. The *t* test values are used to test the null hypothesis that the group means are identical. For brevity, *t* test statistics and the electrode positions associated with the best five ranked feature indices are shown in Table 1. It can be observed that the channel number 4 (F8) corresponding to the right frontal position of brain is assigned the highest rank and the corresponding *t* test statistic is the largest, indicating maximum difference in the mean value of the SE features of both groups. The right occipital region of the brain is assigned the second highest ranking as indicated by the *t* test statistic. This result is very important clinically as it reflects the impact of alcohol on the visual ERP signal of alcoholic subject while performing a visual working memory task. Figure 6 shows specific electrode positions on the scalp associated with the best five ranks.

**Table 1:** Electrode positions of the first five ranked channels and their corresponding *t* test statistic

Ranked channel index	*t* test statistic	Electrode position	Location
4	10.1	F8	Right frontal
30	9.9	O2	Right occipital
15	8.34	T7	Left temporal
33	4	AF8	Right intermediate region between fronto-parietal and frontal
36	2.7	FT7	Left fronto-temporal

The number of ranked/non-ranked channels (N) is chosen arbitrarily as 61 (all), 25 and 15. As far as the pcs are considered, the maximum number is limited by the value of *N*, i.e., if *N* = 25, maximum number of pcs = 25 and below that an arbitrarily chosen numbers of 15 and 5 are considered. The result plots show a smooth interpolation between the arbitrarily selected numbers of pcs for both ranked/non-ranked cases.

**Fig. 7:**
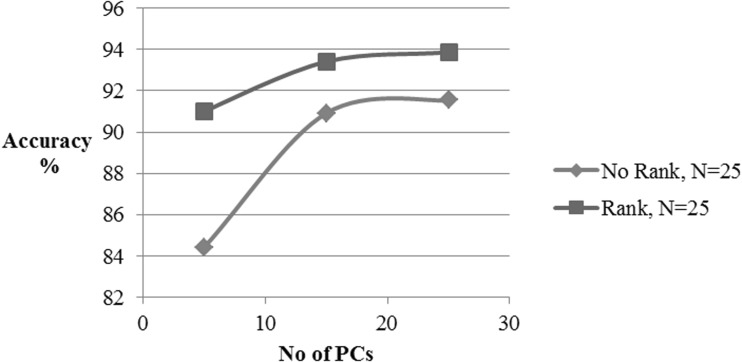
k-NN classifier performance for alcoholic data

**Fig. 8:**
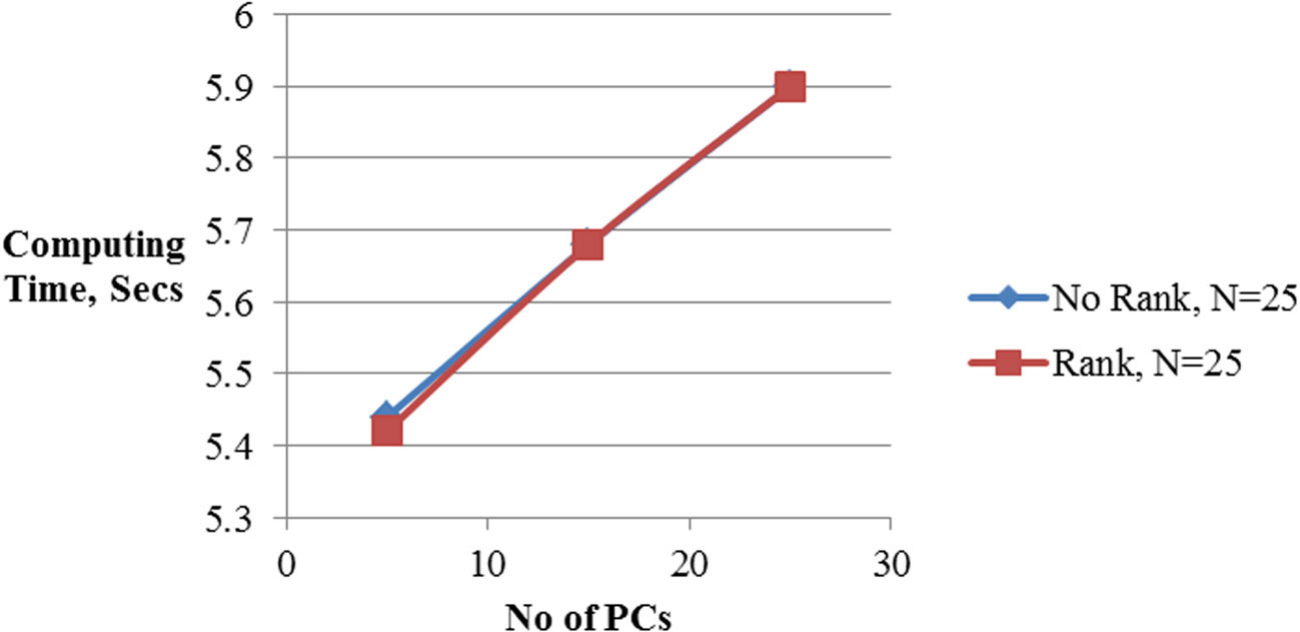
Computation time using k-NN classifier for alcoholic data

**Fig. 9:**
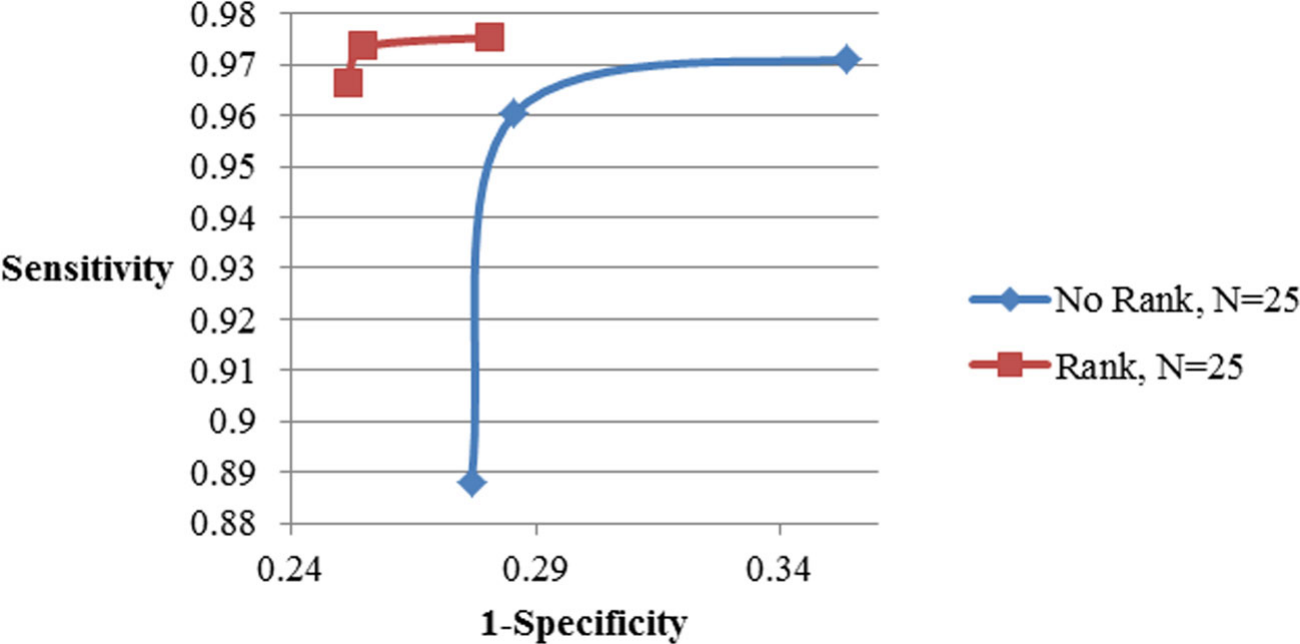
ROC curve using k-NN classifier for alcoholic data

Figures 7, 8, and 9 show the effect of number of pcs on the classification accuracy, computation time, and receiver operating characteristic (ROC) curve for both ranked and non-ranked cases, with *N* = 25. In this case, proposed ranking method improves the k-NN classification accuracy from 91 to 93.87% as the number of pcs increases from 5 to 25. With same number of pcs, the k-NN classifier responds with accuracies of 84.42–91.54% with non-ranked features. Similarly for *N* = 15 and number of pc varying from 5 to 15, ranking enhances k-NN detection accuracies from 88.9 to 93.08% as compared to 86.75–91.96% without ranking (Refer Table 2). Any number of pc ≥ 15 results in an improvement by approximately 1%. With 5 and 15 pcs, the detection accuracy is increased by 6.5 and 2.8%, respectively, for *N* = 25, whereas it enhances by 2.2 and 1%, respectively, for *N* = 15 in comparison with non-ranked features. Also, the minimum number of pcs required to achieve a classification accuracy of above 90% is 15 and 11 for *N* = 25, 15, respectively. The ranking method preserves its superiority for pc greater than 5 in the case of *N* = 15 and *N* = 25. The PCA reduced dimension of the ranked set represents an effective feature subset of statistically significant features to be applied to a k-NN classifier for evaluation of ranked features.

**Table 2:** k-NN classifier performance for alcoholic data

No. of PC	No rank				Rank			
	Efficiency (%)	Sensitivity	Specificity	Time (s)	Efficiency (%)	Sensitivity	Specificity	Time (s)
N = 61								
5	92.21	0.9461	0.7102	5.51	92.21	0.9461	0.7102	5.51
15	94.17	0.9620	0.7316	5.58	94.17	0.9620	0.7316	5.60
25	95.04	0.9820	0.7330	6.10	95.04	0.9820	0.7330	6.05
45	95.40	0.9878	0.7344	6.40	95.40	0.9878	0.7344	6.45
61	95.50	0.9881	0.7346	6.75	95.50	0.9881	0.7346	6.91
N = 25								
5	84.42	0.8881	0.6463	5.44	91.01	0.9662	0.7194	5.42
15	90.92	0.9603	0.7144	5.68	93.42	0.9734	0.7451	5.68
25	91.54	0.9712	0.7232	5.9	93.87	0.9753	0.7480	5.90
N = 15								
5	86.75	0.9091	0.6803	5.42	88.90	0.9201	0.7011	5.44
15	91.96	0.9763	0.7244	5.67	93.08	0.9701	0.7424	5.67

## Discussion

Figure 7 represents the classification accuracy that improves with an increase in the number of pcs of ranked features compared to their non-ranked counterparts. This indicates that the pcs of ranked channels have more information for classification of alcoholics than that of non-ranked set of channels. Figure 8 represents the computation time required for the classification in both ranked and non-ranked cases as a function of the number of pcs. As can be seen, ranking does not impose any limitation on computation time. Figure 9 represents the receiver operating characteristic (ROC) for the classifier in both cases of ranked and non-ranked channels. A typically good ROC always shifts to the leftmost corner. This will ensure that there will be more true-positive instances than the false-positive cases. As expected the ranked case has its ROC pushed toward the left top corner than the non-ranked case.

A channel with first rank will obviously be the best channel as it possesses maximum information regarding the discrimination between alcoholics and controls. In the proposed study, F8 channel is seen to be the best. This result confirms with the findings in the literature that during target stimuli, there will be a gamma band power deficit in alcoholics especially in the frontal lobe. Interestingly, second rank is achieved by O2 channel (associated with the occipital region with the visual cortex underneath). This result correlates with the visual object recognition task used as stimulus while recording EEG in the database under consideration. In fact, these two results are claimed to be the important findings of this study. The accuracy and computation undoubtedly increases with increase in number of pcs. The pcs of ranked channels have better information for classification than their non-ranked counterparts.

All the classification accuracies shown are with respect to 50% holdout cross-validation. The holdout validation method is simple to perform that ensures faster computation. Irrespective of ranking or non-ranking of features, the computation time remains same. This shows that there is no additional computational overhead as far as the classification of ranked channels is concerned. Due to this, the overall CPU time can be greatly reduced while handling large EEG datasets. Also, the computation time consists of the total processor time required for ranking the channels, dimensionality reduction using PCA and classification. In our earlier study [26–29], other robust classifiers such as MLP, SVM, and probabilistic neural network (PNN) were used for the classification of SE and parametric features extracted from the gamma band visual ERP’s of the same alcoholic/control EEG dataset. Even though the PNN classifier responds with excellent classification accuracy close to 100%, it does not generalize as good as other classifiers, and the execution time is proportional to the size of the training set [41]. Due to this, it requires large memory and a more representative training set. The current study used supervised k-NN classifiers for a preliminary assessment of ranked features. In the proposed study, the artifact-free datasets are used and hence the results obtained in the current study are claimed to be more precise and accurate.

The results obtained in the proposed study are clinically important with respect to the impact of alcohol on the visual ERPs of alcoholics and controls. The high ranked channels are located in the frontal, fronto-parietal, temporal, and occipital regions of the brain. In particular, the perception and cognitive processing of a visual stimulus is said to evoke potentials in fronto-parietal and occipital regions of the brain [5]. The results reflect the discriminatory nature of the behavioral sensory control, attention, memory, emotion, and vision of alcoholics with respect to their control counterparts. Interestingly, the channel associated with occipital region is ranked second (Table 1) indicating the influence of alcohol on visual ERP signal produced while performing an object recognition task. A further investigation may be beneficial in studying these aspects in alcoholic patients and find the underlying neural mechanism associated with alcoholism and alcoholic dependence.

A comparison of proposed method with previous studies (for the same EEG dataset) in literature is shown in Table 3. Even though the accuracies and the number of reduced PCA features in this study may not be as impressive as those in previous studies especially that published by the same authors [21], the results can be useful in understanding the contribution of pcs of the ranked channels to enhance the class separation and hence help in the localization of effects of alcohol on different regions of the brain. This assists in identifying regions of brain in which the neuronal activities are highly influenced by alcoholism and alcoholic dependence that lead to cognitive deficits.

**Table 3:** Comparison of proposed method with previous studies using same EEG dataset

Sl. no	Feature selection method	Average classification accuracy		No. of selected channels		Avg. comp time in s	
	Existing methods	NN	FA	NN	FAM	NN	FAM
1	Spectral ratios of *δ* to *γ* band (7 spectral ratios) + GA + NN + FAM classifiers [22]	94.3	81.8	7	7	(Train + 200 test vectors classification time only)	
						0.3	0.17
2	*γ* sub-band power + PCA + k-NN classifier [23]	NN				Not discussed	
		95.83		61			
		94.06		16			
		86.01		8			
		75.13		4			
3	Mean *γ* power and correlation coefficient measure between channels + SVM classifier [24]	80		45		Not discussed	
4	Nonlinear feature extraction (Hurst, Lyapunov exponent, higher-order spectra, ApEn, SaEn) + SVM classifier [25]	91.7		7		Not discussed	
5	Spectral entropy features + SEPCOR + k-NN + MLP classifier [21]	Correlation threshold	Classification accuracy k-NN	Classification accuracy MLP	SEPCOR feature vectors	Computation time (s) k-NN	Computation time (s) MLP
		0.1	99.60	93.43	22	7.30	28.55
				95.45			30.74
				97.55			32.55
				99.60			55.70
		0.08	99.30	89.26	15	5.22	28.31
				91.11			30.07
				93.35			30.04
				95.60			53.60
6	Proposed method spectral entropy features with *t* test ranking +PCA + k-NN + MLP classifier	No of pc. = 25					
		No rank	k-NN		25	k-NN	
			91.54			5.90	
		Rank	93.87			5.90	
		No of pc. = 15					
		No rank	91.96		15	5.67	
		Rank	93.08			5.67	

In previous studies involving the same EEG dataset, the eye blink artifacts were rejected online causing a loss of information. In this study, artifact-free EEG datasets are used in which the artifacts are removed using ICA. This results in more precise classification results as only artifact-free EEG data epochs are presented to the proposed algorithm. In our previous studies, the SE feature extraction was implemented on an unprocessed dataset [26–29] with motion and muscle artifacts. The EEG epochs containing eye blink artifacts were rejected leading to loss of information whereas in our work published later [21] and in the proposed study, artifact-free EEG alcoholic/control datasets are used.

## Conclusion

This paper proposes a robust method to statistically rank SE features in a 64-channel EEG recording for the identification of visual ERP’s produced in the brain that are highly discriminative of alcoholics and control groups. The method uses SE features computed on gamma sub-band visual ERPs of a 64-channel alcoholic/control EEG recording. The proposed statistical *t* test ranking method uniquely identifies channels with maximal separability between class means. Further evaluation of top ranked features is done by applying PCA to subsets of various sizes. As the number of pcs is increased, the classification accuracy improves with ranking. Ranking and reducing of features allow only the best features to be used for classification and hence may provide better generalization.

The effect of pcs on the performances of k-NN classifier is well exploited in the proposed study using ranking and non-ranking procedures. Previous studies have explored several feature selection methods in gamma sub-band range for the identification of alcoholics using the same database. However, the effectiveness of features in the identification of channels with maximum class separation has not been explored in all these studies. The proposed method weighs each feature in terms of its capability to maximize the separation between class means. Also the use of ICA to separate cranial muscle activity (>30 Hz) and motion artifact result in a more valid EEG dataset for processing.

The k-NN classifier takes almost the same amount of time for computations irrespective of ranked or non-ranked channels. The results obtained are clinically significant as the frontal, temporal, and occipital regions of the brain score higher ranks in terms of the SE information for discriminating alcoholics and controls. In particular, the second rank associated with the occipital region directly correlates with the visual object recognition task while recording the EEG data under study. These results may also help in understanding the underlying cortical functions of the selected (ranked) regions of the brain in alcoholic patients. It may also help in reducing the number of channels required in EEG recording of alcoholics. The future work lies in validating these results with a different alcoholic EEG database. The proposed ranking method may also be applied to other time and/or frequency domain features and evaluated using other robust classifiers such as SVM and MLP.

Finally, the proposed study strongly suggests that the pcs of top ranked channels directly correlate with those regions of the brain eliciting reduced responses in gamma range causing cognitive and memory deficits in alcoholics. This may help in exploring the impact of alcohol on visual ERPs.
